# Tooth Autotransplantation in Contemporary Dentistry: A Narrative Review of Its Clinical Applications and Biological Basis

**DOI:** 10.3390/jcm14176249

**Published:** 2025-09-04

**Authors:** Aida Meto, Kreshnik Çota, Agron Meto, Silvana Bara, Luca Boschini

**Affiliations:** 1 Department of Dentistry, Faculty of Dental Sciences, University of Aldent, 1007 Tirana, Albania; kristiankremako@hotmail.com (K.Ç.); agron.meto@ual.edu.al (A.M.); 2 Department of Surgery, Medicine, Dentistry and Morphological Sciences with Interest in Transplant, Oncology and Regenerative Medicine, University of Modena and Reggio Emilia, 41125 Modena, Italy; 3 Department of Dental Research Cell, Dr. D. Y. Patil Dental College and Hospital, Dr. D.Y. Patil Vidyapeeth, Pimpri, Pune 411018, Maharashtra, India; 4 Department of Surgery, Faculty of Dental Medicine, University of Medicine, 1005 Tirana, Albania; silvana.bara@umed.edu.al; 5 Department of Clinical and Experimental Medicine, University of Foggia, 71122 Foggia, Italy

**Keywords:** tooth autotransplantation, biological tooth replacement, periodontal ligament preservation, alveolar bone preservation, oral surgery, endodontics, orthodontics, regenerative dentistry, dental implants alternative

## Abstract

**Background/Objectives**: Tooth autotransplantation is a natural tooth replacement method that preserves the periodontal ligament, supporting root development and alveolar bone remodeling. Unlike dental implants, autotransplanted teeth maintain sensory function and adapt better to the mouth. Although once overlooked, new surgical, imaging, and regenerative advances have revived interest in this technique. This narrative review explores the renewed interest in tooth autotransplantation by assessing its benefits, success rates, technological advancements, and role in modern dentistry while evaluating its advantages, limitations, and potential impact on dental care. **Methods**: A narrative approach was used to provide a comprehensive and descriptive overview of current knowledge on tooth autotransplantation. A literature search was conducted in PubMed, Scopus, and Google Scholar using keywords such as “tooth autotransplantation”, “biological tooth replacement”, “periodontal ligament”, and “dental implants alternative”. English-language articles published between 2000 and 2025 were included, covering clinical trials, reviews, and relevant case reports. Selection focused on studies discussing biological mechanisms, clinical techniques, technological advances, and treatment outcomes. **Results**: Success rates range from 80% to 95%, with better predictability in younger patients with immature donor teeth. Long-term viability depends on preserving the PDL and performing atraumatic extractions. However, challenges such as root resorption, ankylosis, and appropriate case selection remain significant considerations. Technological advancements, including CBCT, 3D-printed surgical guides, and biomimetic storage media, have improved surgical precision and clinical outcomes. **Conclusions**: Tooth autotransplantation is an effective and cost-effective alternative to dental implants, particularly for growing patients or when implants are not suitable. While success depends on surgical skill and proper case selection, improvements in imaging and regenerative techniques have made outcomes more predictable. Future advances in bioengineering, AI-based planning, and regenerative therapies are likely to expand their use in modern dentistry.

## 1. Introduction

**Figure 1:**
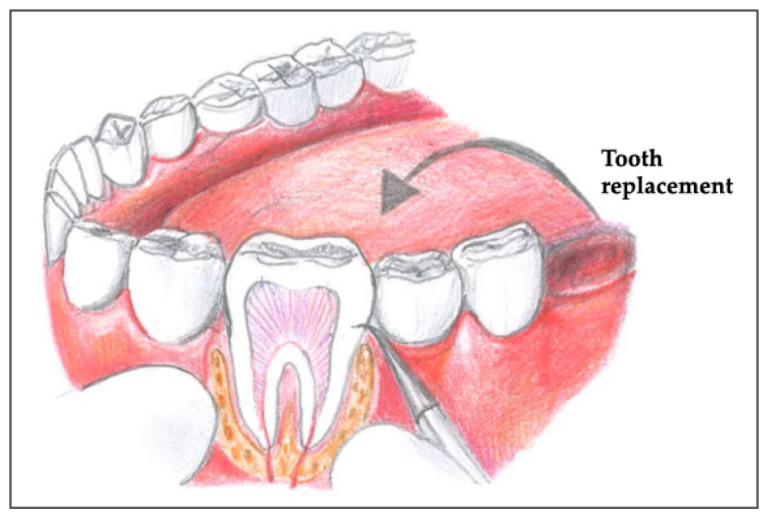
This figure illustrates a dental autotransplantation, highlighting the affected tooth, where the arrow indicates the process of its extraction and subsequent replacement in the missing space. The illustration provides a detailed view of the tooth structure, surrounding gums, and neighboring teeth to emphasize the surgical procedure. Illustration by Nensi Kallfani.

In recent years, the use of dental implants has become increasingly popular. Despite its potential, tooth autotransplantation has been neglected over time. Dental implants have become the predominant solution for tooth replacement in adults due to their high success rates and structural stability. Nevertheless, tooth autotransplantation remains an underutilized but clinically valuable approach, especially in growing individuals or when implants are contraindicated [1,2]. This biologically integrative technique allows for the transplantation of a healthy tooth, commonly a premolar, a canine, or a third molar, to manage an edentulous site, restore a compromised tooth, or relocate an impacted tooth [3], offering advantages like natural proprioception, continued root development, the possibility of orthodontic movement, and preservation of alveolar bone (Figure 1).

The concept of tooth autotransplantation dates back to ancient civilizations. Historical records from ancient Egypt reveal that slaves were often forced to donate their teeth for transplantation, primarily to serve the needs of pharaohs [4]. Over time, the practice of transplanting teeth between different individuals, known as allotransplantation, became more widespread but was eventually abandoned due to complications [4]. Ambroise Paré’s records from 1594 describe tooth transplants, especially among royal families, for restorative or aesthetic purposes. The concept of modern autotransplantation came into focus in the mid-20th century. The modern clinical foundation was established in the mid-20th century when Hale (1954) documented the first standardized case [4].

Subsequent contributions by pioneers such as Dr. Jens Ove Andreasen helped formalize protocols and improve outcomes through systematic case documentation. These advancements laid the groundwork for today’s evidence-based approach to autotransplantation, transitioning it from historical curiosity to a biologically integrated treatment modality [5,6]. In the late 18th and early 19th centuries, tooth transplantation became popular in Europe, especially in England. In London, specialist dentists often performed transplants between individuals, though the procedure was both trendy and controversial [7].

These challenges directed attention toward autotransplantation, a technique in which a person’s own tooth is relocated to another site within their mouth [8,9]. The concept of modern autotransplantation came into focus in the mid-20th century. In 1954, M.L. Hale reported the first well-documented case, laying the foundational principles that continue to guide the procedure today. After this milestone, the 1950s brought more documented clinical cases, marking a time when the approach gained popularity and became part of dental practice [5].

Moreover, pioneers like Dr. Jens Ove Andreasen played a crucial role in advancing dental autotransplantation, carefully recording successful cases. His work not only highlighted the potential of this technique but also contributed to the development of modern methodologies, standardized protocols, and evidence-based practices, making the procedure more effective and improving patient outcomes over time [6,7].

Advancements in cell biology and surgical techniques over the past decades have significantly improved the success of autotransplantation, making it a reliable option for specific tooth replacements [10].

The success of this procedure depends on key factors, including selecting the right donor tooth, preparing the recipient site to match the donor tooth’s root size and shape, and preserving the periodontal ligament (PDL) during explant surgery and transplantation. This technique is most commonly used in young patients with growing jaws, particularly when dental implants are unsuitable, even though it is also a reliable approach in adult patients with a very high long-term success rate [11]. Achieving good functional and aesthetic results requires careful planning, skilled surgery, and proper follow-up care [1]. Artificial dental treatments have progressed because implants offer more reliable results and long-term stability. Unlike natural tooth transplants, which can lead to infection, immune rejection, and root resorption, dental implants stabilize within the jawbone, reducing complications. This makes them a preferred choice for tooth replacement, especially in cases where autotransplantation is not possible [12].

Although structured as a narrative review, this work synthesizes the contemporary scientific literature to integrate evidence-based clinical practice in dental autotransplantation. It examines the biological principles, clinical indications, surgical techniques, outcomes, and recent innovations associated with the procedure. The goal is to provide clinicians with a comprehensive, practice-oriented framework for effective clinical decision-making.

## 2. Methods

This narrative review was conducted to assess the clinical relevance and evolving applications of tooth autotransplantation. A focused literature search was performed using PubMed, Scopus, Web of Science, and Google Scholar with keywords including “tooth autotransplantation”, “biological tooth replacement”, “periodontal ligament”, and “dental implants alternative”.

English-language publications from 2000 to 2025 were included, encompassing clinical trials, case reports, reviews, and observational studies. Studies were selected based on their discussion of biological mechanisms, clinical protocols, technological innovations, and long-term outcomes related to autotransplantation. Given the heterogeneity of study designs and results, a systematic review approach was deemed unsuitable.

Instead, the narrative format enabled a comprehensive and integrative synthesis of foundational concepts alongside emerging advancements such as cone-beam computed tomography (CBCT) imaging, 3D printing, regenerative endodontics, and biomimetic storage media. Emphasis was placed on identifying clinically relevant insights and providing practical guidance informed by current evidence and expert consensus.

## 3. Biological Basis and Histological Mechanisms of Tooth Autotransplantation

**Figure 2:**
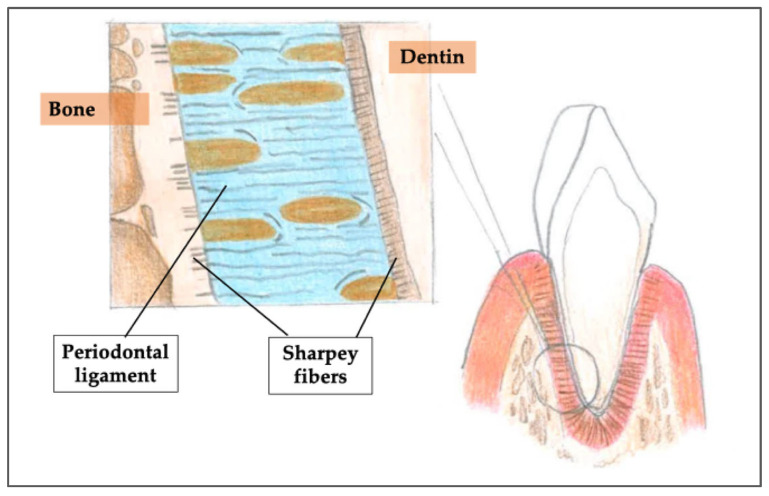
This illustration depicts the tooth anchored to the bone by the PDL, with Sharpey fibers connecting the dentin and cementum to the bone. Illustrated by Nensi Kallfani.

Briefly, the biological success of tooth autotransplantation depends on the tooth’s natural regenerative capabilities and its surrounding structures. The key factor is preserving the PDL, containing multipotent cells that help the tooth’s healing, integration, and long-term viability [13,14]. This specialized connective tissue anchors the tooth to the bone and supports the regenerating bone, cementum, and surrounding tissues after transplantation (Figure 2).

Histologically, the PDL is composed of a dense network of collagen fibers combined with fibroblasts, cementoblasts, osteoblasts, and undifferentiated mesenchymal cells. During autotransplantation, preserving the viability of these cells is crucial. Damage or drying of the PDL can hinder reattachment and cause issues like root resorption or ankylosis. To maintain cell viability, the donor tooth must be extracted carefully and stored temporarily in suitable solutions like Hank’s Balanced Salt Solution (HBSS) or saline. These solutions support the PDL cells’ activity, aiding in the tooth’s regeneration and integration into its new socket [15].

In addition to the proper management of the periodontal tissue, during dental autotransplantation, the pulp tissue must also be correctly managed in order to promote its regeneration, or, at the very least, to prevent complications. Immature teeth with open apices can revascularize effectively due to Hertwig’s epithelial root sheath (HERS): histologically, a bilayered epithelial structure that guides root development and supports odontoblast formation [16]. These odontoblasts produce dentin and maintain pulp vitality, enabling long-term success. In contrast, mature teeth with closed apices lack this regenerative ability and often necessitate root canal treatment to prevent complications due to pulp necrosis and ensure the durability of the transplanted tooth over time.

Bone healing and integration are crucial after placing the donor tooth in the prepared socket, where the alveolar bone remodels in response to the mechanical forces exerted by the tooth. Osteoblasts from the surrounding bone actively form new bone, supported by the PDL. The PDL acts as a cushion to absorb functional stresses and releases growth factors like platelet-derived growth factor (PDGF), vascular endothelial growth factor (VEGF), and transforming growth factor-beta (TGF-β). These factors promote bone regeneration and blood vessel growth, ensuring successful integration [14].

The biomechanical environment within the recipient socket has a significant influence on the healing response. Adequate stabilization of the transplanted tooth is essential to maintain its position and allow the functional reattachment of the PDL. Overly rigid fixation can impair natural remodeling, while excessive mobility may increase the risk of root resorption or displacement. Thus, controlled stabilization that permits limited physiological movement supports optimal outcomes [14,17].

Overall, the histological and biological basis of tooth autotransplantation emphasizes the synergistic relationship between PDL integrity, pulp–dentin regeneration, and alveolar bone remodeling. These natural processes contribute to a more biologically adaptive outcome compared to synthetic alternatives such as implants [16,18].

## 4. Root Resorption in Tooth Autotransplantation

Damage to the PDL can compromise the success of tooth autotransplantation, starting an external root resorption, a destructive process involving clastic cell activity that leads to the loss of root structure, ultimately threatening the stability and longevity of the transplanted tooth [1].

Under normal conditions, the PDL serves as a critical barrier, preventing direct contact between the root and osteoclasts, i.e., cells responsible for resorbing mineralized tissues [19,20]. Once this barrier is damaged, the underlying dentin becomes exposed, causing a cascade of biological responses that lead to tissue degradation [21,22,23].

Mechanical trauma during tooth explantation, excessive orthodontic forces, or improper handling of a donor tooth can disrupt this structure [24].

### 4.1. Types of External Root Resorption: Autotransplantation-Related and Its Pathophysiology

External root resorption is a pathological process that can compromise the outcome. Although multiple types of external root resorption are classified, only three are relevant to dental autotransplantation based on etiology, clinical behavior, and histological features: surface resorption, inflammatory resorption, and replacement resorption (ankylosis) [19,20].

#### 4.1.1. Surface Resorption

Surface resorption is the most common and least severe form. It is a self-limiting, reversible process that typically resolves without intervention. This condition is usually caused by minor external trauma, causing slight injury to the outer layer of cementoblasts [19]. As a result, small surface irregularities or shallow concavities, often less than 0.1 mm, develop on the root surface, making detection difficult on radiographs [19]. These lesions are typically caused by irritation to the PDL and cementum, exposing the underlying dentin.

The exposed dentin activates phagocytic cells that remove the damaged tissue over a 2–3-week period. Subsequently, regenerative cells from adjacent PDL tissues repopulate the area, promoting cementum repair and reattachment of the PDL fibers [19]. This regenerative capacity underscores the importance of maintaining PDL viability during tooth handling and transplantation.

#### 4.1.2. Inflammatory Root Resorption

Inflammatory root resorption is an aggressive, destructive process that typically follows severe damage to the PDL or pulpal infection. When the root surface is exposed and inflamed, multinucleated clastic cells begin to degrade the cementum and dentin [21]. Following injury, inflammatory mediators such as interleukin-1 (IL-1), tumor necrosis factor-alpha (TNF-α), prostaglandins, and matrix metalloproteinases (MMPs) are released at the site. These molecules recruit osteoclast precursors, promoting their differentiation into active osteoclasts that attach to the root and resorb hard tissues [19,25].

If inflammation persists or becomes compounded by microbial contamination, as can occur with poor aseptic technique or infection during transplantation, the resorptive process accelerates. Bacterial infiltration exacerbates cytokine release, intensifying osteoclastic activity and increasing the extent of root damage [23].

Common predisposing factors include prolonged extraoral time, dehydration of PDL cells, and infection of the receiving socket [1,21].

#### 4.1.3. Replacement Resorption (Ankylosis-Related Resorption)

Replacement resorption, also known as ankylosis-related resorption, occurs when the transplanted tooth becomes fused directly to the alveolar bone, bypassing the normal regeneration of the PDL. In this condition, bone is deposited directly onto the root surface, gradually replacing the dental root with osseous tissue [19]. Over time, this fusion results in the loss of physiological mobility and often presents with aesthetic and functional complications, especially in growing patients [20].

This irreversible condition typically arises from extensive PDL damage, excessive pressure during stabilization, or inadequate functional mobility that prevents natural remodeling [19,20]. Ankylosis is particularly problematic in young patients, where continued skeletal growth may lead to infraocclusion and misalignment.

### 4.2. Clinical Implications

All three forms of resorption present unique clinical challenges following tooth autotransplantation. While surface resorption typically resolves with no intervention, inflammatory and replacement resorption can significantly impair prognosis.

Inflammatory root resorption can be supported by contamination of the pulp tissue. Without timely intervention, often requiring endodontic treatment and infection control, this form of resorption can lead to significant structural loss and eventual tooth failure.

Therefore, meticulous surgical technique, atraumatic extraction, the preservation of PDL integrity, minimal extraoral time, and careful post-operative monitoring are essential to reduce the risk of root resorption and ensure the long-term success of the autotransplanted tooth [1,12,19,20,21].

### 4.3. Factors Contributing to Root Resorption

Table 1 presents the key factors influencing root resorption in tooth autotransplantation. Proper management of these factors can enhance the success of the procedure.

## 5. A Step-by-Step Surgical Protocol for Tooth Autotransplantation

Briefly, tooth autotransplantation is a surgical technique that offers a biologically integrative approach to tooth replacement by preserving natural tooth structures and supporting periodontal health. Success depends on precise steps, from choosing the donor tooth to post-transplant stabilization [26].

### 5.1. Donor Tooth Selection

The process begins by selecting a suitable donor tooth, usually from the same quadrant, to match the morphology and dimensions of the recipient site [27]. Third molars are commonly used for replacement. Ideally, the donor tooth should have an incomplete root formation with an open apex (over 1 mm) to support blood flow and healing [28]. For teeth with fully formed roots, transplantation remains viable if endodontic treatment is performed either before or immediately after the procedure [11,29], although new trends suggest waiting for the appearance of clinical or radiographic signs before proceeding with treatment [30,31].

### 5.2. Preparation of the Recipient Site

Next, the recipient site is prepared before extracting the donor tooth, when it is possible. This involves modifying the socket of the extracted tooth to fit the donor tooth by reshaping it, often removing the intra-alveolar septum in case of a multirooted socket, or creating a new surgical socket. The preparation could be performed using low-speed burs with saline irrigation to prevent heat damage. Proper preparation ensures a suitable fit and minimizes trauma to the surrounding tissues, avoiding compression between the root of the donor tooth and the receiving socket to preserve the viability of the PDL [26,32,33].

The preparation should be hands-free as well as surgically guided with customized osteotomes or driven drills planned on CBCT and 3D scans [2,31]. The use of 3D techniques seems to reduce complication rates and improve long-term survival.

### 5.3. Atraumatic Explantation of the Donor Tooth

The donor tooth is carefully explanted with minimal trauma to preserve the PDL cells, which are essential for successful transplantation. During this process, the tooth is temporarily stored in a biocompatible medium, such as HBSS or saline-soaked gauze, to maintain PDL cell viability and prevent desiccation [34,35]. Additionally, Casaroto et al. (2010) studied propolis extract as an alternative storage medium for avulsed teeth [36]. Their findings showed that propolis effectively preserved PDL cell viability, which was comparable to HBSS, the gold standard. This suggests propolis could be a biocompatible and accessible option when conventional storage media are unavailable.

### 5.4. Insertion and Stabilization of the Donor Tooth

The explanted donor tooth is inserted into the prepared recipient site at the correct orientation and position, slightly below the occlusion plane, to prevent trauma. To avoid the latter, tooth stabilization is achieved using splinting materials, such as wires or composite resin, to secure the transplanted tooth. A semi-rigid splint is applied for a period of 2 to 4 weeks, allowing controlled physiologic mobility that supports PDL reattachment and bone remodeling. During this period, regular occlusal assessments are crucial to monitor the tooth’s position, detect any signs of mobility, and make necessary adjustments to prevent unfavorable movement. Proper stabilization, combined with careful post-operative monitoring, plays a key role in ensuring the long-term success of the autotransplanted tooth [12].

### 5.5. Patient and Case Selection for Optimal Outcomes

The success of tooth autotransplantation depends on selecting the right patient (Table 2). Ideal candidates are healthy, with good oral hygiene and no systemic conditions that may impair healing. The donor tooth must be healthy and placed in a clean, infection-free recipient site with adequate bone support. Additionally, anatomical compatibility is important. The crown must fit within the available space in the arch, and the root shape should allow for extraction. Straight and parallel roots (in the case of multirooted teeth) are ideal, whereas apical curvatures and divergent roots would complicate the procedure. Rapid handling and minimal time outside the mouth are critical for preserving the PDL cells and ensuring reattachment [1].

Tooth autotransplantation has well-established indications in pediatric and adolescent patients, offering distinct biological and developmental advantages over dental implants or prosthetics [37]. In growing individuals, the preservation of the PDL supports continued alveolar bone development, maintains proprioceptive function, and allows for orthodontic adaptability, factors that are particularly critical during craniofacial maturation.

(A) **Pediatric-Specific Indications**

- Traumatic Tooth Loss: Frequently observed in children and adolescents, especially in anterior regions. Autotransplantation of premolars can restore function and esthetics while preserving alveolar bone growth.
- Congenitally Missing Teeth: Commonly involving lateral incisors or second premolars. Premolar autotransplantation serves as a viable alternative to implants, which are contraindicated during skeletal growth.
- Ectopic and Impacted Teeth: Impacted canines and other teeth can be surgically repositioned using autotransplantation, particularly when orthodontic traction is not feasible or would require prolonged treatment.

(B) **Surgical Protocol Considerations**

- Immature Roots with Open Apices (>1 mm): Preferred because they enhance revascularization and pulpal healing.
- Minimal Extraoral Time and Atraumatic Extraction: Essential for maintaining PDL viability and supporting continued root development.
- Avoidance of Rigid Fixation: Semi-rigid splinting is recommended to allow for physiological mobility and prevent ankylosis, which is particularly detrimental in growing patients.

### 5.6. Prevention and Management

Preventing and managing root resorption in tooth autotransplantation requires careful attention to surgical techniques, biological preservation, and post-operative care. Addressing the factors that contribute to root resorption can significantly improve the long-term outcomes [31]. These strategies can be categorized into prevention measures and management strategies when resorption is detected, as seen in Table 3. In cases where resorption occurs, endodontic treatment, biocompatible materials, and surgical interventions can help preserve the tooth and improve the clinical results.

## 6. Types of Cases in Tooth Autotransplantation

**Figure 3:**
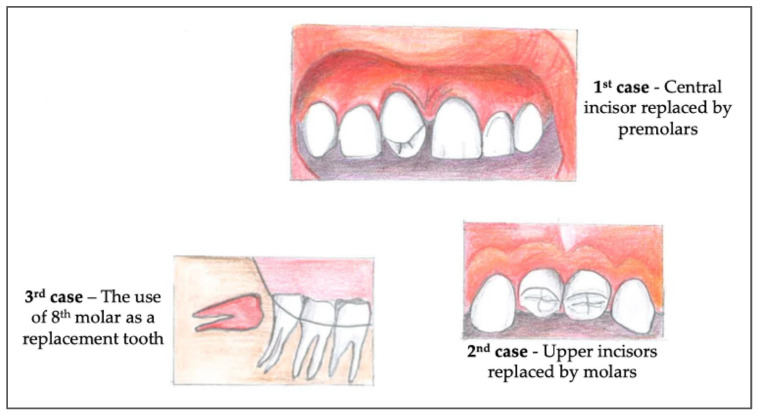
Various clinical cases of tooth autotransplantation. Illustration by Nensi Kallfani.

Figure 3 highlights common situations where tooth autotransplantation can be a useful and effective option, depending on the specific needs of each clinical case.

### 6.1. Replacement of Missing or Extracted Teeth

**Figure 4:**
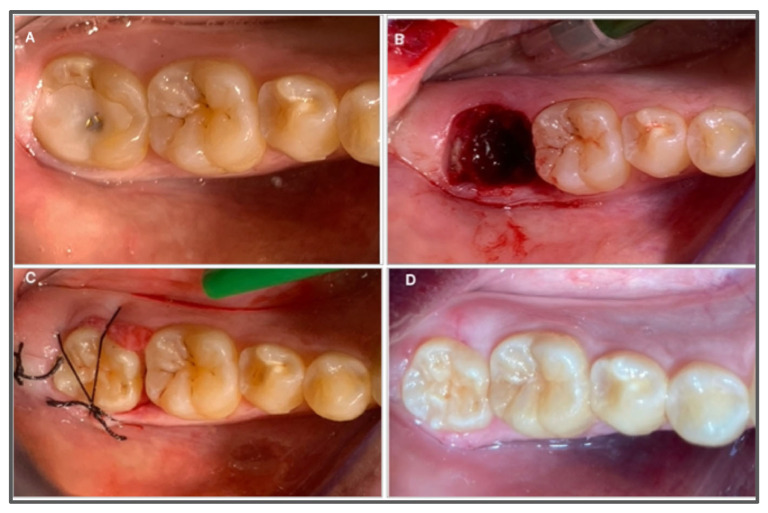
Clinical images illustrating the step-by-step process of third molar autotransplantation to replace a compromised second molar. (**A**) Pre-operative condition showing the compromised second molar and the available omolateral impacted third molar as the donor’s tooth. (**B**) The recipient site, after extraction of the second molar, revealed the prepared socket. (**C**) The third molar was positioned into the extraction socket and stabilized with sutures. (**D**) One-month post-transplantation image after suture removal, demonstrating successful healing and integration of the transplanted tooth.

One of the most common uses of tooth autotransplantation is to replace missing or extracted teeth, especially molars. Third molars are often chosen as donor teeth, particularly those with incomplete root development. This is beneficial for young patients, as it helps preserve the alveolar bone and dental function (Figure 4).

### 6.2. Management of Traumatic Tooth Loss

Autotransplantation is also effective for replacing teeth lost due to trauma, especially in children and adolescents. For example, premolars can be transplanted into the anterior region to restore both function and esthetics after an incisor is lost in an accident or sports injury. This approach is particularly beneficial in preserving the alveolar ridge and supporting long-term dental development.

### 6.3. Treatment of Congenitally Missing Teeth

For patients with congenitally missing teeth (such as lateral incisors or second premolars), autotransplantation provides a natural, cost-effective alternative to implants or prosthetics. Premolars are often selected as donor teeth, offering a good match in size and shape for anterior replacement. This method is ideal for younger patients whose jaws are still growing.

### 6.4. Orthodontic Space Closure and Malocclusion Management

Tooth autotransplantation can also play a role in orthodontic treatments, especially for closing gaps or correcting malocclusions. Premolars that are extracted as part of orthodontic treatment can be transplanted to replace missing teeth, maintaining the integrity of the dental arch. Additionally, impacted or misaligned teeth can be repositioned through autotransplantation, avoiding the need for extended orthodontic procedures.

### 6.5. Replacement of Non-Restorable Teeth

For severely damaged teeth, such as those affected by extensive decay or fractures, autotransplantation offers a solution when conventional restoration is not possible. This approach helps preserve alveolar bone and natural tooth function, and the transplanted tooth can continue to develop biologically, providing a long-term solution.

### 6.6. Adjunct to Regenerative Procedures

In cases with significant bone loss or periodontal disease, tooth autotransplantation can be combined with regenerative techniques like platelet-rich plasma (PRP) or bone grafts. These therapies help support bone healing and ensure the transplanted tooth remains stable, especially when the recipient site lacks sufficient bone volume.

### 6.7. Management of Impacted or Ectopically Positioned Teeth

**Figure 5:**
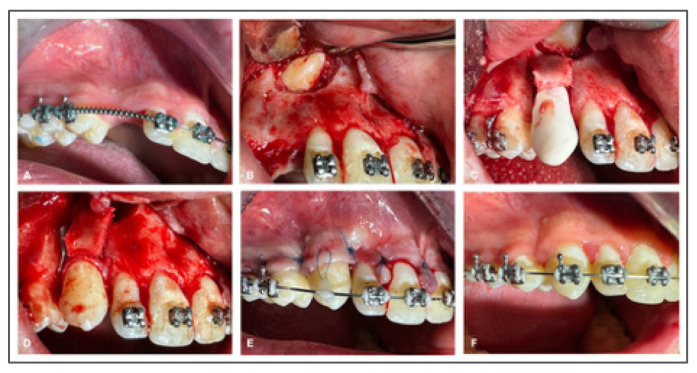

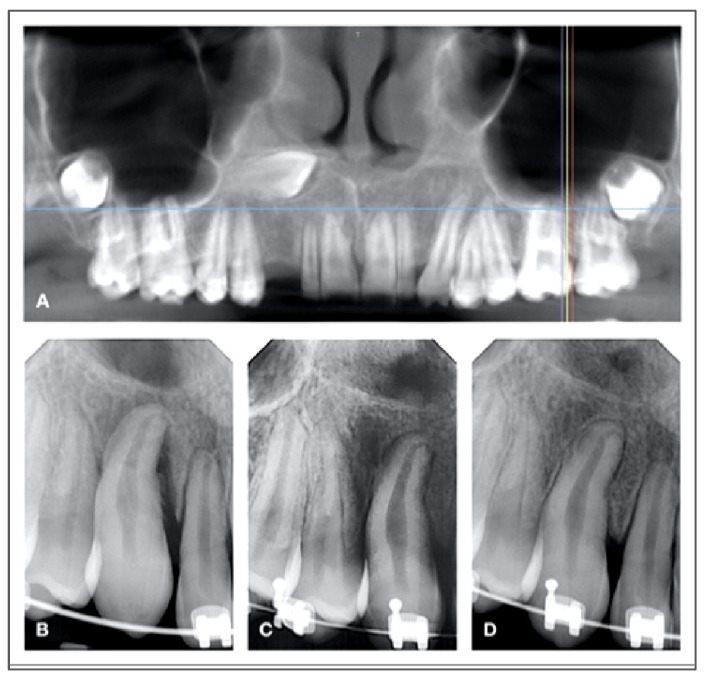
The (**upper**) panel illustrates the clinical sequence of an autotransplantation procedure involving an impacted tooth (tooth #1.3). (**A**) Pre-operative condition showing the initial orthodontic alignment with space maintained for the transplantation. (**B**) Surgical exposure of the impacted canine crown. (**C**) Preparation of the recipient site by splitting the alveolar crest and using a donor tooth replica to shape the socket. (**D**) Placement of the transplanted tooth into the prepared site. (**E**) Flap closure with sutures and stabilization of the transplanted tooth using composite to secure it within the arch. (**F**) Post-operative healing observed one year later, with continued orthodontic treatment to ensure proper alignment and integration of the transplanted tooth. The (**lower**) panel consists of radiographic evaluations supporting the autotransplantation of an impacted maxillary canine (tooth #1.3). (**A**) Pre-operative panoramic radiograph showing the impacted canine, which was erupting into the nasal cavity. (**B**) The immediate post-operative periapical radiograph confirms the transplanted canine’s positioning in its new socket. (**C**) Radiographic follow-up at three months, monitoring initial healing and stabilization. (**D**) One-year follow-up radiograph demonstrating complete healing and successful integration of the transplanted tooth within the alveolar bone.

Tooth autotransplantation is also useful for impacted or ectopically positioned teeth that cannot be aligned orthodontically. For example, impacted maxillary canines or third molars can be repositioned into their ideal position through autotransplantation, providing a faster resolution than traditional orthodontics (Figure 5, upper and lower panels).

## 7. Clinical Decision-Making Approach for Case Selection

**Scheme 1:**
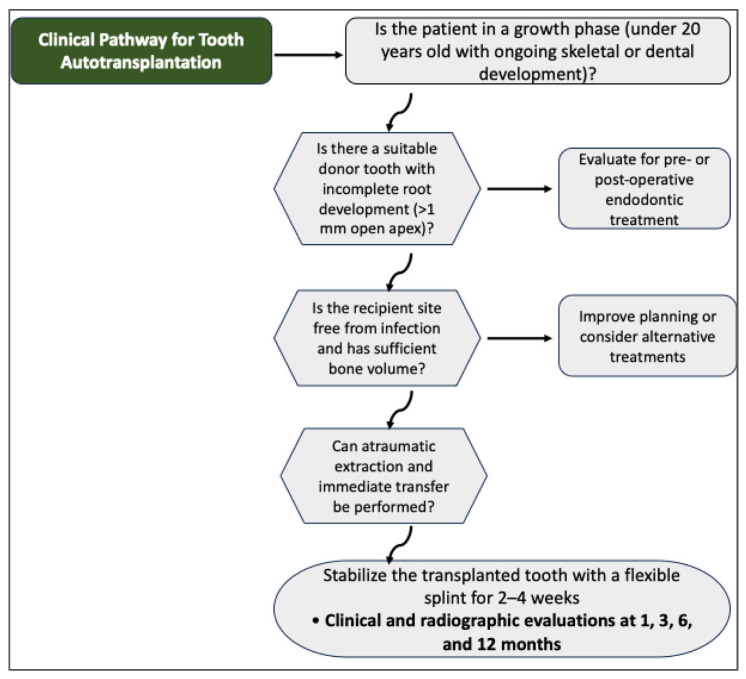
This flowchart guides clinicians in evaluating whether a patient is a suitable candidate for tooth autotransplantation. It outlines a sequence of decision points, starting with patient age, donor tooth development, and recipient site condition, leading to clinical action. The diagram emphasizes atraumatic technique, proper planning, and follow-up care to support successful outcomes and consistent clinical practice.

While various clinical scenarios have demonstrated the applicability of tooth autotransplantation, proper case selection remains the core of success. To assist clinicians in planning, a simplified decision-making sequence is presented in Scheme 1. This flowchart outlines the key considerations for determining case suitability based on patient factors, donor tooth status, and recipient site readiness.

### 7.1. Classification and Timing of Dental Autotransplantation

Dental autotransplantation procedures can be classified based on the timing of tooth placement in relation to the extraction or avulsion occurrence [38,39]:

- Immediate autotransplantation is performed directly following tooth extraction or avulsion, before significant alveolar remodeling has occurred.
- Delayed autotransplantation typically takes place within 15 to 20 days post-extraction, allowing initial healing while maintaining socket architecture.
- Late autotransplantation involves transplanting a tooth after complete healing of the recipient site. In such cases, the socket must be surgically reconstructed (alveoloplasty), often supplemented by orthodontic splinting to ensure stability and alignment.

Late autotransplantation may present greater challenges due to alveolar ridge resorption and socket atrophy, which can compromise recipient site integrity and complicate donor tooth anchorage. Hence, the time interval between explantation and transplantation plays a critical role in determining procedural complexity and prognosis.

Autotransplantation is a time-sensitive intervention reserved for cases where the PDL remains viable [40]. Ideally, the procedure should be completed as fast as possible, and the tooth must be adequately preserved after explantation during the surgical phases. The choice of storage medium significantly affects PDL cell survival:

- Dry environment: ~18 min of viability;
- Saline: <2 h;
- Milk: up to 3 h;
- HBSS: up to 24 h (considered the gold standard in the United States).

The primary objective of immediate tooth autotransplantation is to reestablish the biological continuity of the PDL, enabling full regenerative healing (restitutio ad integrum) [41]. This happens when clinical intervention is performed within a narrow surgical time window.

The success of the procedure relies on a carefully structured protocol:

1. Donor Tooth Selection: A detailed evaluation of both the receiving site and the donor tooth anatomy is performed to assess anatomical compatibility and identify any contraindications.
2. Explantation: A gentle explantation is mandatory, avoiding PDL damage; an orthodontic pre-treatment should be performed to simplify the procedure because it causes tooth mobility.
3. Tooth Repositioning and Splinting: The tooth is reinserted into the socket with minimal pressure and stabilized using a semi-rigid splint. It is crucial that the splint permits slight physiological mobility and does not exert tension on the tooth, which could disrupt reattachment. The recommended duration of splinting varies:
  ○ One week, as per Andreasen’s protocol.
  ○ Two–three weeks according to Tsukiboshi’s guidelines.
  During this period, pulp vitality and signs of healing are closely monitored.
4. Gingival Suturing: Soft tissue margins are approximated and sutured to reestablish mucogingival continuity and protect the surgical site.
5. Post-Operative Radiography: A periapical radiograph is obtained immediately after reimplantation to confirm accurate repositioning and stabilization.
6. Pharmacologic Support: Post-operative management includes topical antiseptics (0.2% chlorhexidine mouthwash for 30 days) and systemic antibiotics for 7 days to minimize infection risk and support periodontal healing.

This structured approach enhances the likelihood of favorable biological integration and minimizes complications such as inflammatory root resorption or ankylosis.

### 7.2. Endodontic Considerations and Healing Dynamics

The necessity and timing of endodontic treatment after tooth autotransplantation depend largely on the stage of root development at the time of avulsion:

- Teeth with complete root development typically require root canal therapy within two weeks post-reimplantation. This is often accompanied by the placement of an interim calcium hydroxide dressing, which promotes an antimicrobial environment and aids in monitoring periodontal healing. To delay the root canal treatment, waiting for a better stabilization of the transplanted tooth, an extraoral apicoectomy has been suggested during the surgery, because it is a fast technique that allows an immediate seal of the canals [42]. New trends propose not to treat mature transplanted teeth, suggesting potential healing [30,31].
- Teeth with incomplete root formation (open apex) are monitored for signs of pulp revascularization. If reinnervation fails to occur or if clinical or radiographic indicators of inflammatory root resorption develop, endodontic treatment becomes necessary to preserve the tooth and surrounding structures.

This tailored approach helps optimize tissue healing and long-term outcomes, especially in younger patients with greater regenerative potential.

### 7.3. Age-Dependent Risk of Root Resorption

Root resorption following reimplantation exhibits age-related variability due to differences in metabolic activity, bone remodeling rates, and regenerative capacity [38,43]. The approximate timelines for expected resorption are shown in Table 4.

### 7.4. PDL Healing: Reattachment vs. New Attachment

The success of autotransplantation largely depends on PDL healing, which can occur through two primary mechanisms:

- Reattachment involves the realignment of existing PDL fibers on the root surface with those in the alveolar socket. This process can occur rapidly, within two weeks, and restores up to two-thirds of the original biomechanical integrity of the periodontium. This type of healing is possible in cases of post-extraction transplantation in the areas of the recipient site that do not require remodeling and if there is proximity between the socket wall and the root surface.
- New attachment takes place when PDL remnants are only partially viable. In such cases, regenerative healing is required, characterized by the formation of new cementum and PDL fibers to restore attachment. This healing is slower and more vulnerable to disruption.

If the root surface is extensively damaged or the tooth is non-vital, regeneration may be impaired, predisposing the site to inflammatory resorption and potential failure.

### 7.5. Surgical Techniques

Atraumatic Extraction of the Donor Tooth: This procedure involves the careful removal of the donor’s tooth using instruments like periotomes, elevators, or piezoelectric tools. This minimizes damage to the root surface and surrounding tissues, ensuring the viability of the PDL cells essential for healing and reattachment.

Recipient Site Preparation: The recipient site must be prepared to fit the donor’s tooth. Low-speed burs are used under saline irrigation to avoid thermal damage to the bone. Proper alignment within the socket ensures the donor’s tooth functions well and reduces the risk of complications such as ankylosis or root resorption.

Handling and Storage of the Donor Tooth: If immediate transplantation is not possible, the donor’s tooth is stored in biocompatible solutions like HBSS, milk, or saline-soaked gauze. These preserve the metabolic activity of the PDL cells, maintaining their viability until the tooth can be transplanted.

Tooth Positioning and Stabilization: The donor tooth is placed slightly below the occlusal plane to avoid trauma during healing. Stabilization is achieved with flexible splints made from orthodontic wires or composite resin, which allow limited mobility. This supports PDL remodeling and reattachment.

Post-Operative Care and Monitoring: Regular follow-up visits are essential to monitor the healing process and check for any complications, such as root resorption or infection. Radiographic imaging, including CBCT, is used to assess revascularization and integration of the transplanted tooth.

### 7.6. Technological Advancements

Furthermore, the success of tooth autotransplantation relies on precise surgical techniques and advanced technologies that enhance procedural accuracy and outcomes. These approaches minimize complications, improve biological integration, and expand the indications for this procedure. Below is a detailed exploration of the main surgical techniques and recent technological innovations that have transformed tooth autotransplantation (Table 5).

## 8. Success Rates of Tooth Autotransplantation vs. Dental Implants

Tooth autotransplantation and dental implants are both effective solutions for replacing missing teeth, but their success rates depend on various factors such as patient conditions, clinical scenarios, and procedural specifics.

### 8.1. Success Rates of Tooth Autotransplantation

Tooth autotransplantation shows high success rates, typically 80–95%, with the best outcomes in younger patients. Success depends on factors such as age, root development, surgical technique, and post-operative care.

Children achieve rates up to 95% due to superior healing, bone remodeling, and open apices that promote revascularization. Transplanted teeth also adapt to jaw growth, unlike implants, which may cause infraocclusion and esthetic issues.

Although complications like root resorption or pulp necrosis may occur, modern techniques have minimized these risks. When properly indicated, autotransplantation provides excellent long-term functional and esthetic results, often outperforming implants in growing patients.

### 8.2. Success Rates of Dental Implants

Dental implants typically have a success rate of 90% to 98%. Their success depends on osseointegration, where the titanium implant bonds with the bone. Factors like bone quality and volume, overall health (e.g., diabetes, smoking, etc.), and proper placement affect the clinical result. Despite this, implants can face issues like peri-implantitis (inflammation) and might need extra procedures, such as bone grafts, adding to the cost and complexity.

### 8.3. Comparison of Success Rates

Both procedures are highly successful, but each is suitable in different situations. Tooth autotransplantation is best for younger patients as it preserves natural tooth structures, helping with bone development. Dental implants are better for adults with fully developed jaws and enough bone support. While implants offer predictable results, they lack the biological integration and sensory response that autotransplantation provides. Complications can arise with each procedure: autotransplantation may lead to root resorption, whereas implants can develop peri-implantitis.

Tooth autotransplantation offers unique biological advantages, such as proprioception, growth compatibility in children, and natural bone maintenance. Unlike implants, which are more susceptible to peri-implantitis and do not adapt to skeletal growth, autotransplanted teeth show better integration in growing patients and exhibit fewer long-term biological complications when PDL preservation is optimal. Nevertheless, the technique requires careful surgical expertise and is less standardized across various practices.

### 8.4. Cost-Effectiveness

Tooth autotransplantation is more cost-effective than dental implants since it avoids costly prosthetics and procedures like bone grafting, making it an affordable biological option.

## 9. Limitations and Future Outcomes of Tooth Autotransplantation

Even tooth autotransplantation, like any dental procedure, has limitations that can affect its applicability and suitability. These factors must be carefully considered when selecting clinical cases and planning the procedure.

### 9.1. Limitations

The following table (Table 6) summarizes the main limitations, followed by an exploration of potential advancements and future outcomes.

### 9.2. Future Advancements

**Scheme 2:**
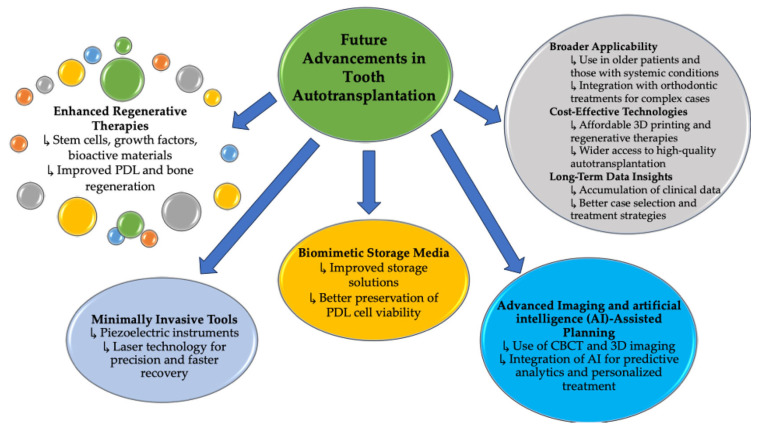
Schematically, some key future advancements in tooth autotransplantation are given, categorized into five main areas: biomimetic storage media for better preservation of PDL cells, advanced imaging and AI-assisted planning for improved diagnostics and treatment, broader applicability and cost-effective technologies to enhance accessibility, minimally invasive tools for precision and faster recovery, and enhanced regenerative therapies to promote better healing and integration.

The future of tooth autotransplantation looks promising, with continuous progress focused on surpassing its limitations and enhancing its use. One of the major areas of progress lies in enhanced regenerative therapies, where the use of stem cells, growth factors, and bioactive materials is expected to significantly improve PDL and bone regeneration, ultimately enhancing the stability and longevity of transplanted teeth. Another important development is the introduction of minimally invasive tools, such as piezoelectric instruments and laser technology, which allow for more precise surgical techniques, minimizing trauma to surrounding tissues and promoting faster recovery. Additionally, advancements in biomimetic storage media are being explored to improve storage solutions for donors’ teeth, ensuring better preservation of periodontal ligament cell viability, which is crucial for successful reattachment and healing. The integration of advanced imaging and AI-assisted planning further enhances the accuracy of the procedure, with CBCT and 3D imaging enabling a precise pre-operative assessment, while AI-driven analytics facilitate personalized treatment strategies and predictive modeling for better patient outcomes. Furthermore, the broader applicability of tooth autotransplantation is being explored, particularly for older patients and individuals with systemic conditions, as well as in combination with orthodontic treatments for complex cases. Cost-effective technologies, such as affordable 3D printing and regenerative therapies, are expected to make high-quality autotransplantation more widely accessible. At the same time, the accumulation of long-term clinical data will refine case selection and treatment protocols, ensuring more predictable results. Scheme 2 includes the probable key developments:

## 10. Discussion

Tooth autotransplantation has regained attention as a biological alternative for replacing missing or non-restorable teeth, offering clear advantages over implants by preserving the PDL, maintaining proprioception, and supporting natural bone remodeling [1,2,20]. These biological benefits allow continued root development and functional adaptation, making the procedure particularly valuable in younger patients where implants may lead to infraocclusion and require future interventions [7,8].

The procedure’s success, reported at 80–95%, depends on careful case selection, donor tooth maturity, surgical technique, and post-operative care [10,20,36]. Donor teeth with open apices have the highest success due to their superior revascularization and continued root formation. Conversely, inflammatory root resorption, ankylosis, and technique variability remain challenges, underscoring the importance of atraumatic handling, minimal extraoral time, and close follow-up [17,44].

Technological innovations have improved both planning and outcomes. CBCT and 3D imaging enable an accurate assessment of donor and recipient sites, while computer-guided surgery and 3D-printed templates enhance surgical precision and reduce operative time [31,45,46]. Adjuncts, such as biomimetic storage media (e.g., HBSS, platelet-rich fibrin), further enhance PDL cell viability and reduce complication risks [34,36].

In pediatric patients, autotransplantation provides unique regenerative and developmental benefits. Unlike implants, transplanted teeth integrate with jaw growth, preserving alveolar bone and occlusion [29]. Nevertheless, compromised PDL healing may lead to ankylosis and infraocclusion, reinforcing the need for long-term monitoring [47]. Successful outcomes also depend on patient compliance, emphasizing the importance of informed consent and multidisciplinary coordination.

When compared with other pediatric options (adhesive bridges, orthodontic space closure, space maintainers), autotransplantation offers a biologically integrated and growth-compatible solution, whereas alternatives provide only temporary or esthetic benefits.

Although CBCT and guided surgery enhance accuracy, their use in children may be limited by cost, accessibility, and concerns about radiation exposure. In such cases, conventional planning still achieves favorable results when executed by experienced clinicians.

Despite high success rates, evidence on long-term outcomes, particularly in pediatric cohorts, remains limited. Future multicenter studies with standardized protocols and long-term follow-up are needed. While the technique is sensitive and limited by donor tooth availability, advancements in regenerative endodontics, AI-assisted planning, and improved PDL preservation are expected to increase predictability and broaden clinical indications. Ultimately, tooth autotransplantation stands out as a reliable, biologically favorable option for both growing and adult patients.

## 11. Conclusions

Tooth autotransplantation is a biologically favorable and clinically effective option for replacing missing or non-restorable teeth, particularly in younger patients and in cases where implants are contraindicated. When combined with proper case selection, precise surgical techniques, and biologically sound protocols, it delivers excellent functional, esthetic, and long-term outcomes.

This technique is especially valuable in managing traumatic tooth loss, congenitally missing teeth, ectopic or impacted teeth, and compromised molars. High success rates in growing individuals are attributed to the preservation of the PDL, continued root development, and natural proprioception, contributing to both clinical success and patient satisfaction.

Advancements in imaging, regenerative endodontics, and biomimetic storage media have further improved predictability and safety. However, challenges remain, including the lack of standardized protocols, dependence on surgical expertise, and limited long-term data, particularly in mature teeth.

With continued research focused on protocol refinement, long-term prognosis, and the integration of AI-assisted planning and regenerative technologies, autotransplantation has the potential to become a routine, cost-effective, and biologically superior alternative to dental implants in appropriately selected cases.

## Figures and Tables

**Table 1:** Factors that can influence root resorption.

Factors	Impact on Root Resorption
Extraoral Time	Prolonged exposure leads to PDL cell death, increasing the risk of resorption. Immediate transplantation or storing the tooth in HBSS or saline during the surgery protects the PDL and reduces this risk.
Explantation Trauma	Using excessive force or improper techniques can damage the PDL and cementum, exposing the root and increasing the risk of resorption. Gentle, atraumatic extraction helps protect these structures.
Recipient Site Conditions	Infections, lack of bone support, or poor socket preparation cause inflammation, raising the risk of resorption. Maintaining aseptic conditions, shaping the socket properly, and ensuring adequate bone volume help prevent this.
Stability of the Recipient Site	Strong stabilization prevents PDL reattachment, while excessive mobility delays healing, increasing the risk of ankylosis or resorption. Proper stabilization ensures successful transplantation.

**Table 2:** Clinical criteria for patient and tooth selection in autotransplantation.

Criteria	Recommendation	Description
Patient Age	Ideal ≤ 20 years	Younger patients have better healing and bone remodeling capacity
Donor Tooth Type	Canine, premolars, or third molars	Must have similar root morphology to recipient site
Root Development	Better incomplete apex (>1 mm open)	Allows revascularization and continued development
Recipient Site	Non-infected, sufficient bone	Adequate volume and hygiene are critical
Alternatives	Implants, bridges	Consider implants for adults with poor donor options

**Table 3:** Prevention and management strategies for root resorption in tooth autotransplantation.

Management Strategies	Prevention Strategies
**Endodontic Treatment**: Use endodontic therapy to remove inflammation and seal defects with biocompatible materials like MTA or calcium hydroxide.	**Preservation of the PDL**: Use atraumatic techniques and preservation media to maintain PDL viability.
**Biocompatible Materials**: Apply MTA or Biodentine to repair resorptive lesions and support tissue regeneration.	**Control of Inflammation**: Use anti-inflammatory agents and antibiotics to reduce inflammation and prevent infection.
**Surgical Interventions**: Use surgical repositioning or GBR for severe cases, or tooth extraction if necessary. Regenerative therapies like PRP or stem cells can enhance healing.	**Stabilization Techniques**: Employ flexible splinting techniques to stabilize the tooth while allowing PDL regeneration.
	**Regular Monitoring**: Use radiographs and cone-beam computed tomography (CBCT) to detect resorption early for timely intervention.

**Table 4:** Estimated root resorption timelines following tooth reimplantation by age group.

Estimated Timeframe for Root Resorption	Age Group
Within ~2 years	Pre-pubertal patients
May extend up to 10 years	Post-pubertal adults
Higher risk of complications including infraocclusion and esthetic concerns	Adolescents

**Table 5:** Main surgical techniques and technological advancements in tooth autotransplantation.

Benefits	Description	Aspects
Ensures viability of PDL cells for reattachment and healing.	Gentle removal of the donor tooth to preserve PDL cells.	Atraumatic Extraction
Prevents complications like ankylosis and ensures proper occlusion.	Shaping the socket to fit the donor tooth dimensions and orientation.	Recipient Site Preparation
Enhances accuracy and minimizes risks to adjacent structures.	3D imaging for detailed pre-operative planning.	CBCT Imaging
Ensures precise socket preparation and donor tooth alignment.	Virtual surgical planning with custom surgical guides.	Computer-Guided Surgery
Reduces intra-operative variability and improves efficiency.	Fabrication of patient-specific surgical templates.	3D Printing
Accelerates bone and soft tissue repair, promoting better integration.	Use of PRP and PRF to enhance healing and tissue regeneration.	Adjunctive Regenerative Therapies
Enhances long-term outcomes by ensuring continued root development.	Techniques to maintain pulp vitality and support root growth in immature teeth.	Regenerative Endodontics
Reduces bleeding, minimizes discomfort, and promotes faster recovery.	Minimally invasive techniques for soft tissue management.	Laser-Assisted Surgery

**Table 6:** Limitations of tooth autotransplantation.

Description	Limitations
Limited to patients with suitable donor teeth and adequate recipient site conditions.	Case Selection Constraints
Reduced regenerative potential and higher risk of complications in older patients.	Age-Related Challenges
Requires high surgical precision and expertise to preserve PDL and ensure proper placement.	Complex Surgical Technique
Includes root resorption, pulp necrosis, infection, and ankylosis, especially in mature teeth.	Complications and Risks
Lack of extensive studies on outcomes over several decades limits knowledge about durability.	Limited Long-Term Data
Advanced techniques and tools may not be available in all clinical settings, restricting their use.	Accessibility of Technology

## Data Availability

The data presented in this study are available on request from the corresponding authors.
